# Heating Dwellings

**Published:** 1874-08

**Authors:** 


### HEATING DWELLINGS.

THERE is only one way to heat dwellings healthfully in the northern States of the Union—open fireplaces and open doors. Very much of what has been said of the unhealthfulness of furnace heat is without any just foundation, and is founded on ignorance of their management, or the unreasonableness of persons who want to heat a large house in midwinter with a scuttle of coal every twenty-four hours; and, to forward their views, every crevice and crack about the doors and windows of the whole building is carefully plugged up, so that not a particle of pure air can come in from without, with the inevitable result that none of the foul air within can escape, while every additional breath drawn by the inmates increases the impurity. The same servant who kindles as large a fire in the grate on a warm, rainy morning as would be done if the thermometer was at zero, is entrusted to the management of the furnace, and to save labor, both of mind and body, instead of replenishing the furnace with a small amount of coal every hour or so, or more or less often, according to the out door temperature, the furnace is heaped up with as much as it can hold three times a day, with the result of letting it cool until the coal is heated and begins to burn well, and then to become unendurably hot for hours together, making it necessary to open the windows, thus causing the useless waste of trying to heat up all out doors. Besides, the furnace becomes red hot, scorches the air, and sends a contaminated atmosphere throughout the whole house.

It is unreasonable to expect an uncultivated servant girl to manage a furnace properly in a climate like ours, which sometimes varies forty degrees or more in half a day. It is the business of the intelligent mistress of the house to keep herself informed of the weather changes, by having a thermometer in doors and out, and give the servant corresponding orders. Sometimes the fire in the furnace burns well, briskly, and gives out a large amount of heat; at other times it will not burn, gives out but little warmth, and the whole household shivers, and then the servant is blamed for neglecting the furnace; the real reason being that the fire will not burn, either because the wind blows in a direction to destroy the draught of the furnace, or because the atmosphere is so heavy that it drives down the chimney. And yet a reasonable amount of intelligent attention may greatly modify these discomforts. If the wind "blows down the chimney," then the passage for the out door air conveying it to the furnace should be more or less fully closed. This tends to compel a draught up the chimney, if the valves at the lower part of the house are kept open. There is not, perhaps, in all New York a full sized house which can be anything like comfortably heated with any ordinary furnace during the dozen coldest days of winter, and instead of attempting to do so, only a part of the rooms should be heated, by concentrating all the heat in them. As this very cold weather does not occur but for a very few days during the whole winter, it is better to adopt this management than to endanger the burning of the building, and to ruin the air for all purposes of healthful breathing, by keeping the furnace at a red heat, in the vain attempt to warm the whole house. The use of weather strips is in the highest degree unwise and mischievous. In the attempt to save a few cents' worth of fuel every day, the whole household is compelled to breathe a foul atmosphere all day long and all night too—the strips excluding the pure air, and to the same extent preventing the free escape of foul air. It is really a matter of surprise that an intelligent person should ever allow a weather strip in a habitable dwelling. If such is the case it is because the subject has not commanded the reflection which was due to it.

